# Bronchoscopic Management of COPD and Advances in Therapy

**DOI:** 10.3390/life13041036

**Published:** 2023-04-18

**Authors:** Benjamin DeMarco, Christina R. MacRosty

**Affiliations:** 1 Division of Pulmonary Diseases and Critical Care Medicine, Department of Medicine, University of North Carolina at Chapel Hill, Chapel Hill, NC 27514, USA; 2 Section of Interventional Pulmonology and Pulmonary Oncology, Division of Pulmonary Diseases and Critical Care Medicine, Department of Medicine, University of North Carolina at Chapel Hill, Chapel Hill, NC 27514, USA

**Keywords:** bronchoscopic lung volume reduction, BLVR, chronic obstructive pulmonary disease, COPD, hyperinflation, emphysema, endobronchial valve, mucus hypersecretion, chronic bronchitis

## Abstract

Chronic Obstructive Pulmonary Disease (COPD) is a highly prevalent and morbid disease marked by irreversible structural changes in the lungs. Bronchoscopic therapies have significantly expanded the treatment armamentarium for patients with persistent symptoms by reducing the physiologic detriments of hyperinflation in a less invasive fashion than surgical lung volume reduction. The spectrum of bronchoscopic techniques to reduce hyperinflation includes endobronchial valves, coils, thermal ablation, and biologic sealants. Other therapies focus on reducing parasympathetic tone and mucus hypersecretion and include targeted lung denervation, bronchial rheoplasty, and cryospray techniques. In this article, we will review the variety of techniques for bronchoscopic lung volume reduction, both established and investigational, along with their respective benefits and complications and will briefly review other investigational therapies for COPD.

## 1. Introduction

Chronic obstructive pulmonary disease (COPD) is a clinical syndrome characterized by structural pulmonary abnormalities, impaired lung function, and resultant chronic respiratory symptoms such as dyspnea, cough, and exercise limitation. COPD has a worldwide prevalence of ~10%, is the third leading cause of death worldwide, and in 2019 was responsible for over three million deaths and 74 million disability-adjusted life years [1,2]. Emphysema is one of the most common structural pulmonary abnormalities associated with COPD. It is characterized by the destruction of alveolar airspaces caused by an inflammation-induced imbalance between protease and antiprotease activity within the lung [3]. These pathologic changes lead to loss of elastic recoil in the lung, early airway closure during exhalation, and resultant air trapping in the distal airspaces. The subsequent hyperinflation pushes the diaphragm downward into a flattened, mechanically disadvantaged position during the respiratory cycle, precipitating breathlessness and exercise intolerance [4]. Hyperinflation has also been linked to cardiac and skeletal muscle dysfunction, further contributing to refractory dyspnea and worsened quality of life [5,6].

Given the variety of negative physiologic and clinical consequences of hyperinflation, as well as the mounting evidence that reducing hyperinflation can improve respiratory symptoms, decrease systemic inflammation, and improve metabolic parameters, reduction of hyperinflation has become a focus of interventional therapy in patients with COPD [7,8]. Standard COPD therapies such as long-acting bronchodilators and pulmonary rehabilitation programs can decrease hyperinflation, but their effects are limited and do not address the underlying mechanical disruption and structural damage seen in advanced emphysema [9,10,11]. Evolving from the evidence proffered by lung volume reduction surgery (LVRS), bronchoscopic lung volume reduction (BLVR) has significantly expanded the treatment paradigm offering a less invasive pathway for patients with emphysema refractory to optimized medical care. The success seen with endobronchial valves (EBV) for bronchoscopic lung volume reduction has led to development of other interventions to treat the physiologic components of COPD including hyperinflation, increased parasympathetic tone, and mucus hypersecretion. This article will review the current evidence for bronchoscopic lung volume reduction therapies including their clinical benefits and respective complications and will briefly discuss other bronchoscopic therapies currently under investigation.

## 2. Bronchoscopic Lung Volume Reduction

### 2.1. History

Bronchoscopic lung volume reduction techniques evolved from benefits demonstrated with lung volume reduction surgery (LVRS). LVRS involves the bilateral wedge resection of 20–35% of each emphysematous lung, accomplished through video-assisted thoracoscopic surgery (VATS) or, less commonly, median sternotomy. The landmark study in LVRS was the National Emphysema Treatment Trial (NETT) which compared lung volume reduction surgery to standard medical therapy in patients with severe emphysema. NETT demonstrated no mortality benefit in the intention to treat the population; however, it did show mortality benefit and symptom burden reduction (measured via Saint George’s Respiratory Questionnaire (SGRQ)) in a subgroup analysis of patients with heterogenous upper-lobe-predominant airspace disease and low exercise capacity [12]. Application to wider patient populations is limited by negligible functional gain and higher morbidity and mortality but provided the foundational evidence prompting investigation into bronchoscopic techniques [13]. One advantage of EBV for BLVR is that, unlike in the surgical literature, BLVR has demonstrated efficacy and safety in patients with homogeneous disease, thus expanding interventional options for patients with persistent symptoms who would not be able to undergo surgical lung volume reduction [14]. Multiple BLVR techniques are available or being investigated, including endobronchial valves, endobronchial coils, thermal vapor ablation, and biologic lung volume reduction (sealants and sclerosants), among others. As of this writing, EBVs remain the only procedure for BLVR that is approved by the US Food and Drug Administration (FDA).

### 2.2. Pre-Procedural Screening and Evaluation

Precise patient selection is key to optimizing benefits and minimizing adverse effects when considering bronchoscopic lung volume reduction. In general, bronchoscopic lung volume reduction therapies should only be considered in patients with significant symptom burden (as assessed by validated questionnaires such as the modified Medical Research Council (mMRC ≥ 2) or COPD Assessment Test (CAT score ≥ 10)) and limitation in exercise performance (6 min walk test (6MWT) distance > 100 m but < 450 m) [15,16]. Medical therapy tailored to Global Initiative for Chronic Obstructive Lung Disease (GOLD) guidelines, abstinence from smoking and participation in pulmonary rehabilitation should be optimized. While minor variation exists, pulmonary function criteria generally include significant airflow obstruction (evidenced by a post-bronchodilator forced expiratory volume in one second (FEV1) of 15–50% predicted) and significant hyperinflation (evidenced by total lung capacity (TLC) ≥ 100% predicted and residual volume (RV) ≥ 175% predicted). Lung volume parameters are ideally measured by body plethysmography to provide the most accurate data. Diffusing capacity for carbon monoxide (DLCO) ≥ 20% predicted is suggested, although this DLCO target is not a definitive exclusion criterion. Measurement of DLCO in advanced emphysema is challenging and often not a true reflection of gas exchange properties, and good outcomes have been published in patients with very low (<20%) DLCO [17,18]. It is important to consider the full spectrum of pulmonary function testing, arterial blood gas sampling, and chest imaging when determining eligibility of patients with low DLCO [17].

Patients with severe resting hypoxemia (PaO2 < 45 mm Hg), hypercarbia (PaCO2 > 50 mm Hg), or pulmonary hypertension (systolic pulmonary artery pressure > 45 mm Hg) are generally excluded given they are at risk for worsening hypercarbia and hemodynamics as a result of changes in ventilation–perfusion relationships after EBV placement and may be better served with transplant evaluation [19,20]. While one small study showed improvement in ventilation–perfusion mismatch after BLVR, further testing is needed to determine whether patients with chronic hypercarbia would benefit [21]. Significant heart failure (left ventricular ejection fraction < 40%) and anticoagulation or antiplatelet therapy that cannot be held peri-procedurally are contraindications due to cardiac and bleeding risks associated with the procedure. Prior thoracic surgery (previous lobectomy, lung transplantation, or lung volume reduction surgery) in the target lobe is a contraindication as the procedure may not be successful in such cases. Prior pleurodesis is a relative contraindication and is dependent on whether pleurodesis was performed on the side of the target for EBV placement. Patients requiring maintenance immunosuppressive agents, prednisone at moderate or high doses, or with frequent infectious exacerbations (chronic bronchitis phenotype or symptomatic bronchiectasis) are generally not eligible due to high risk of local microbiologic colonization of endobronchial devices [22].

Imaging findings play an important role in patient selection for BLVR. Presence of large bullae, incomplete fissures, significant paraseptal emphysema, interstitial lung disease, lung nodules suspicious for malignancy (or those that need to be followed with sequential imaging), and bronchiectasis are contraindications to EBV therapy [19,20,23,24]. Computed tomography (CT) imaging of the chest is the best method for evaluation of pleural and parenchymal abnormalities for patients undergoing BLVR evaluation. Furthermore, CT imaging is used to determine fissure integrity, degree of emphysema, and lobar volume to help with target lobe selection and is discussed in more detail below [20,25,26]. Perfusion imaging may be helpful in patient/target lobe selection, particularly in patients with homogeneous disease. Perfusion can be assessed via planar ventilation/perfusion scintigraphy, which gives general estimates of ventilation/perfusion to zones within each lung (usually divided into upper, middle, and lower zones), or with a single-photon emission computed tomography scan (SPECT-CT), which provides relative lobar perfusion data. Both studies are performed in specially trained nuclear medicine departments and, particularly in the case of planar ventilation/perfusion scintigraphy, are generally available within the US. Our center obtains perfusion imaging in every patient who is undergoing endobronchial valve placement to assist in target lobe selection and to understand lobar perfusion prior to their procedure.

The final step in evaluation for EBV placement is selection of a target treatment lobe. The first recommended step in target selection is visual assessment of a high-resolution CT scan of the chest (HRCT) in order to identify possible target treatment lobes and to identify any concurrent disease that may defer or disqualify a patient for valve treatment (paraseptal emphysema, suspicious pulmonary nodule, bronchiectasis, etc.). Thin-slice (1 mm) noncontrast inspiration and expiration images are recommended with reconstruction in coronal, sagittal, and axial views in order to perform quantitative CT analysis (QCT). QCT can be performed through a variety of commercially available software tools based on a pathologically validated attenuation threshold (950 Hounsfield units) for measuring emphysema [27,28,29]. QCT is essential to characterize the degree of emphysematous destruction of the lungs as too little emphysematous lung in a target lobe will lead to atelectasis of functional lung. Resultant V/Q mismatch, dyspnea, and chest discomfort will hamper the symptomatic benefit of the procedure [22]. QCT will generate a “lobe destruction score” based on the percentage of low-attenuation areas as well as a “fissure completeness score” (FCS). The most common cutoffs for lobe destruction are at least 30% of target lobe > −950 Hounsfield units or at least 50% > −910 Hounsfield units with a slight variation amongst prior clinical trials [12,19,20]. The VENT trial was foundational in identifying patient characteristics most likely to benefit from BLVR, specifically the importance of “complete” fissure integrity (≥90% FCS on HRCT) in achieving sustained outcomes [30]. Fissure integrity is determined using a computer-generated 3D map of all fissures via an algorithm that uses multiple anatomic landmarks near the fissure such as vessels and airways [31]. The absence of fissure integrity is an important surrogate marker for the presence of collateral ventilation (CV), as higher fissure integrity correlates with less chance of CV. Collateral ventilation normally occurs through pores of Kohn and bronchoalveolar communications of Lambert, lower resistance pathways that may be enhanced in emphysema due to airways obstruction. The presence of CV precludes EBV placement as bronchial occlusion and atelectasis will fail due to alternative routes of target lobe ventilation. QCT is sufficient to confirm presence of collateral ventilation based on FCS of <80%; but in FCS between 80% and 90%, it is not specific enough for final target selection and should be combined with direct measurement of CV using the Chartis^™^ Pulmonary Assessment System (PulmonX Inc., Redwood City, CA, USA) [26]. As of this writing, the Spiration Valve System is FDA approved for patients with COPD who meet selection criteria and have FCS of 90% or greater on QCT evaluation. The Zephyr Valve system is approved for EBV therapy in patients with COPD who meet selection criteria and should be used in combination with the Chartis^™^ System for measurement of CV in patients with FCS between 80% and 95%, although Chartis assessment is recommended in patients with FCS > 95%.

The Chartis^™^ Pulmonary Assessment System is a proprietary catheter that measures flow and volume via a sensor located on the distal end of a balloon catheter. The balloon is inflated to occlude the distal airway of the target segment (simulating the effect of an EBV in situ), while the sensor gathers data which are displayed on the attached screen in real time. If no collateral ventilation is present, the target is considered collateral ventilation negative (CV-), and airflow from the target lobe will gradually decrease. Continuous flow measured with the flow catheter indicates the presence of collateral ventilation and the lobe is termed collateral ventilation positive (CV+). HRCT and Chartis^™^ have been validated via intraoperative fissure assessment with 76% and 71% accuracy, respectively [32]. HRCT has demonstrated higher sensitivity while Chartis^™^ has higher specificity. When used together, however, all cases of incomplete fissure integrity were detected in the validation studies [26,32]. These authors recommend routine performance of Chartis^™^ assessments to ensure expected treatment benefit. This also confirms that any lack of volume reduction after EBV placement is likely due to valve misplacement or other factors rather than collateral flow, a practice that is shared by other expert centers [22,33]. Furthermore, use of the Chartis^™^ system during the procedure allows the treating physician to assess clinical response to target lobe occlusion; if the patient experiences hypoxia or other complication during lobar occlusion with the flow catheter, they may not tolerate placement of EBV despite all pre-procedure inclusion criteria being met. Selecting the optimal target lobe requires synthesis of all diagnostic information. The ideal target lobe is characterized by high emphysematous heterogeneity (unless the patient has homogeneous disease), balanced lung volumes in the ipsilateral nontarget lobe, lower relative perfusion, and lack of parenchymal or pleural features likely to limit atelectasis. Both the Chartis^™^ measurement and the placement of EBV, if indicated by lack of CV, are typically performed in a single procedure to minimize procedural and anesthetic time. General anesthesia is recommended for EBV placement, no matter which device is being used, in order to reduce coughing and allow precise measurement of airway diameter for valve sizing, although 14–35% of the procedures performed in clinical trials used conscious sedation with no substantial difference in outcomes when comparing the two anesthetic strategies [19,20,34,35]. Once the absence of collateral ventilation has been confirmed, either with pre-procedure QCT alone or with QCT and direct measurement, the valves are placed in all subsegments of the target lobe under direct vision. Airway anatomy will dictate the number of valves required; generally, three to five are placed per treatment.

Given the significant logistics, cost, necessary expertise, and narrow therapeutic range of BLVR, it is strongly recommended that bronchoscopic treatment strategies for patients with severe COPD be discussed in a multidisciplinary forum with participation from individuals with expertise in obstructive lung disease pulmonology, interventional pulmonology, thoracic surgery, and lung transplant. At our center, this multidisciplinary group conducts a comprehensive assessment of the patient’s history, pulmonary function tests, chest radiology, thoracic perfusion studies, echocardiography, and blood gas testing before making recommendations about additional necessary testing and BLVR candidacy. Furthermore, a multidisciplinary team approach ensures that patients who do not meet criteria for BLVR are referred for other potential therapies which may be of benefit, such as LVRS or lung transplant evaluation. The multidisciplinary team approach ensures a systematic and organized evaluation to optimize patient selection and that the best possible treatment is offered to each individual patient. A growing evidence base specific to BLVR supports this multidisciplinary approach [36,37].

### 2.3. Endobronchial Valve Placement

**Figure 1:**
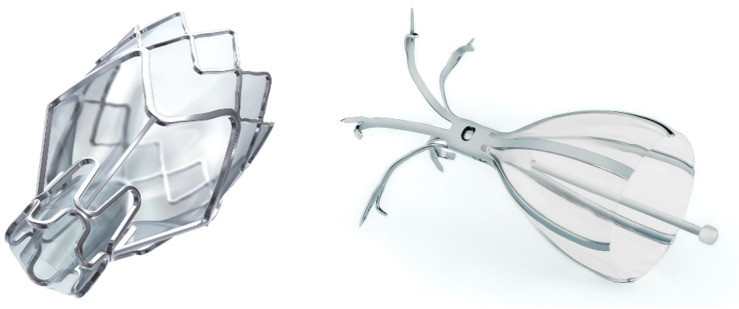
**Left**: Zephyr^®^ endobronchial valve (image courtesy of PulmonX Inc., Redwood City, CA, USA); **right**: Spiration Valve System^®^ (image courtesy of Olympus, Tokyo, Japan).

EBVs are the mainstay of treatment with a robust evidence base and are the only FDA-approved device for bronchoscopic lung volume reduction. EBVs are placed into the carefully selected target lobe and act as one-way valves in order to allow air to escape the target lobe during expiration but preclude air from entering during inspiration. The result is lobar atelectasis. This lobar atelectasis achieves lung volume reduction via reduction in residual volume and improvement in diaphragmatic excursion with subsequent improvement in lung function parameters, increased exercise performance, and improved quality of life [14,19,20,23,35,38,39]. Table 1 summarizes the clinical trial results that lead to precise definition of selection criteria and consistently improved outcomes with EBV insertion. The two FDA-approved EBVs are the Zephyr^®^ endobronchial valve (PulmonX Inc., Redwood City, CA, USA) and the Spiration Valve System^®^ (Olympus, Tokyo, Japan) shown in Figure 1.

The Zephyr^®^ EBV is a self-expanding Nitinol (a nickel-titanium alloy) frame supported by a silicone membrane that causes atelectasis of the target lobe via a duckbill-shaped one-way outlet. There are four sizes available including 4.0 EBV (4.0–7.0 mm airway diameter range and 6.9 mm sealing length), 4.0-LP EBV (4.0–7.0 mm airway diameter range and 5.2 mm sealing length), 5.5 EBV (5.5–8.5 mm airway diameter range and 8 mm sealing length), and 5.5 LP EBV (5.5–8.5 mm airway diameter range with 5.8 mm sealing length). Zephyr valves are deployed via flexible bronchoscopy with a proprietary deployment catheter with airway sizing markers for determination of airway diameter and length [30,40]. The distal end of the Zephyr valve is seated on an airway carina to prevent migration.

**Figure 2:**
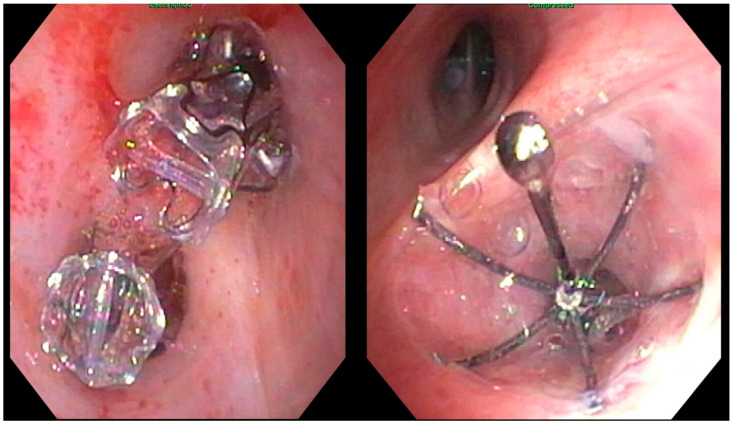
Endobronchial valves in situ; **left**: Zephyr^®^ endobronchial valve; **right***:* Spiration Valve System^®^ (images supplied by the authors).

The Spiration Valve System^®^ is composed of a nitinol strut skeleton covered in a polyurethane polymer membrane. Upon bronchoscopic deployment into the airway through the proprietary deployment catheter, the nitinol struts act as stabilizing anchors, and the valve assumes an umbrella shape. This structure minimizes contact with surrounding tissues and facilitates the clearance of distal airflow and mucus during expiration. Airways are sized with a proprietary balloon sizing catheter in order to determine which of the four available valve sizes (5, 6, 7, or 9 mm) should be deployed [20,41]. Both EBV types are shown after placement in Figure 2.

### 2.4. Post-Procedural Outcomes

While the VENT trial demonstrated statistically but not clinically significant improvements in FEV1, 6MWT, and SGRQ, post hoc analysis defined the importance of emphysematous heterogeneity (defined as difference in emphysema destruction score by QCT between target lobe and the ipsilateral lobe of >15%), complete lobar occlusion, and complete fissure integrity as strong predictors of therapeutic response [30,40]. The subsequent IMPACT, TRANSFORM, STELVIO, and BeLieVer-HiFi studies were designed specifically to enroll heterogeneously emphysematous patients without collateral ventilation. These studies demonstrated improvement in lung function and exercise capacity to varying degrees [14,23,35,38]. The IMPACT and TRANSFORM populations have 12 and 24 month follow-ups, respectively, with durable benefit demonstrated in lung function, quality of life, exercise capacity, and BODE index [42,43]. The LIBERATE and EMPROVE trials utilizing the Zephyr^®^ EBV and Spiration Valve System^®^, respectively, were the pivotal trials leading to FDA approval of EBVs for BLVR, demonstrating improvements in FEV1, 6MWT, and SGRQ [19,20]. A recent meta-analysis of nine studies with 1300 patients demonstrated statistically significant improvement in FEV1, 6MWT, and SGRQ compared to the standard of care that persisted for at least six months in patients with heterogenous emphysema and no CV [44].

Adverse events associated with EBV placement are most common in the early post-treatment period (summarized in Table 2). Respiratory complications occurred in 31 to 35% of patients in the treatment arm, while only 5 to 12% of patients in the placebo arm experienced complications [14,19,20,23,30,35,38,39]. A course of prophylactic antibiotics and steroids are sometimes prescribed periprocedurally in order to decrease incidence of respiratory exacerbation or pneumonia. This practice varies locally and is largely pragmatic given the lack of evidence base. Pneumothorax is the most serious adverse event and often requires tube thoracostomy to manage. Pnuemothoraces most frequently occur in the ipsilateral lobe due to redistribution of volume as the untreated lobe expands in compensation for volume reduction of the target lobe, leading to a defect in the visceral pleura [45]. Most reported pneumothoraces occurred with 72 h after EBV placement, and thus a three-day inpatient stay for monitoring is recommended after the procedure in addition to conservative measures such as minimizing elevated airways pressures, treating cough with cough suppressants, and minimizing activities that cause increased intrathoracic pressure [46]. This risk for pneumothorax persists up to 45 days, and patients should be educated on associated symptoms and when to seek medical care [19,45]. Late complications leading to loss of valve efficacy such as valve migration, mucus impaction, or granulation tissue formation have been described with revision rates up to 41% [45,47].

## 3. Investigational Therapies for BLVR

### 3.1. Endobronchial Coils

After endobronchial valves, endobronchial coils are the most-studied treatment modality for bronchoscopic lung volume reduction. Lung volume reduction coils (LVRC), produced primarily by PneumRx^®^ (Mountain View, CA, USA), are shape-memory nitinol. Local practice may vary, but in general each treatment involves the placement of 8–14 coils (sizes 100, 125, or 150 mm) under general anesthesia with fluoroscopic deployment guidance. The patients are hospitalized for observation following the procedure, and each additional target lobe is treated sequentially in future procedures separated by 4–6 weeks [48]. The coil design compresses surrounding diseased tissue in order to restore tissue tension to emphysematous areas and subsequently reduce air trapping and improve elastic recoil [48,49,50]. In addition, coils may reduce airflow in target segments leading to redistribution towards less obstructive regions of the lung. They are “non-blocking” devices and thus can be effective in patients with interlobar collateral ventilation. RESET (n = 47, UK), RENEW (n = 315, USA/Europe), and REVOLENS (n = 100, France) are the largest randomized controlled trials examining nitinol coils compared to conventional medical care for severe emphysema. Each found, with varying follow-up times, a statistically significant improvement in 6MWT as well as smaller improvements in FEV1 and quality of life measured by SGRQ compared to usual care [51,52,53]. Outcomes did not differ between heterogenous and homogenous emphysema. A post hoc analysis of the RENEW trial demonstrated that patients with baseline residual volume > 200%, emphysema score > 20% (based on quantitative CT analysis), and absence of airway disease were more likely to have clinically meaningful improvements in lung function and quality of life [49]. These findings prompted the ELEVATE trial, designed to validate this patient criteria prospectively [54]. In the aforementioned trials, the complication rate was higher in the intervention group (coils) compared to the conventional therapy group. Most common complications in the coil group were COPD exacerbation, pneumonia, and pneumothorax. In the largest of the three, RENEW, major complications (including hospitalization and other potentially life-threatening or fatal events) occurred in 34.8% of coil participants vs. 19.1% of usual care. LVRC are a recognized potential therapy in the 2023 GOLD Report for the Diagnosis, Management and Prevention of Chronic Obstructive Lung Disease, but clinical availability, number of complications, and paucity of long-term clinical outcomes data limit their current application and warrant further study [55].

### 3.2. Thermal Vapor Ablation

Bronchoscopic thermal vapor ablation (BTVA) is accomplished via instillation of heated water vapor to a target pulmonary segment in order to induce a local inflammatory reaction. The reaction triggered by delivery of this thermal energy causes scarring, fibrosis, and eventual volume loss in order to reduce hyperinflation. Because of the local inflammatory response following the procedure, BTVA is not recommended in patients with known alpha-1-antitrypsin deficiency, asthma, chronic bronchitis, or bronchiectasis, and patients with cardiovascular or pulmonary vascular disease should be carefully reviewed [56]. Additionally, patients should be monitored closely after the procedure in order to detect complications and proactively monitor for symptoms of the localized inflammatory response. The vapor dose is calculated based on the volume and density of the targeted lung tissue to be treated via a proprietary software (Uptake Medical Corporation, Seattle, WA, USA). The earliest multicenter study involved 44 patients with upper-lobe predominant emphysema and demonstrated improvement in lung function (17% increase in FEV1) and quality of life (14-point reduction in SGRQ) [57]. The first randomized controlled trial of BTVA versus medical management (STEP-UP) used a segmental bilateral treatment strategy. Individual segments were targeted based on the extent of disease [58]. The thermal vapor ablation arm demonstrated a 14% increase in FEV1 and nine-point reduction in SGRQ compared to the conventional treatment arm, but there was not significant difference in 6MWT or RV between the two groups at six months [58]. In post hoc analysis, these outcomes were not affected by interlobar fissure integrity or the presence or absence of collateral ventilation [58]. The treatment group experienced higher rates of complications including COPD exacerbation (24 vs. 4%) and pneumonia (18 vs. 8%). The data for BTVA remain sparse, and this therapy should only be considered within clinical trials in patients with upper-lobe-predominant heterogenous emphysema (with or without collateral ventilation).

### 3.3. Biologic Lung Reduction

The foundation of biologic lung reduction is bronchoscopic instillation of a substance that induces an inflammatory reaction with subsequent remodeling of lung parenchyma, formation of fibrosis, and contracture in order to achieve lung volume reduction. A variety of materials have been explored including sealants, adhesives, and autologous blood [59,60,61]. The details of the various material properties, benefits, and limitations are outside the scope of this review but are well described elsewhere [61]. The most well studied is a hydrogel sealant product known as AeriSeal^®^ or emphysematous lung sealant (ELS) (PulmonX Inc., Redwood City, CA, USA) that is instilled bronchoscopically via a catheter into the targeted airway. After nonrandomized trials showed promising results, a randomized control trial was initiated (ASPIRE) but terminated early for non-regulatory reasons after randomization of only 95 patients such that the primary 12-month endpoint could not be assessed [60,62,63]. The treatment group demonstrated high morbidity (43% requiring hospitalizations) and significant mortality (two deaths) [62,63]. Biologic lung volume reduction is not currently recommended, and further randomized controlled trials are needed to examine efficacy and safety of ELS.

### 3.4. Airway Bypass Stents

The Exhale^®^ airway bypass procedure (Broncus Technologies, Mountain View, CA, USA) is based on the insertion of specialized stents placed endobronchially in order to facilitate emptying of emphysematous lung and lead to lung volume reduction. The stents are coated in silicone and elude paclitaxel in order to maintain patency. This is performed with a specialized catheter that creates bronchial fenestrations that are maintained by the drug eluting component of the stents [33]. One randomized trial has examined the placement of these stents and included patients with homogenous emphysema, FEV1 ≤ 50% predicted, and RV ≥ 150% predicted; 208 patients were treated and 107 underwent a sham procedure. This study demonstrated early improvement in lung function and dyspnea at 1 month; however, this improvement was not durable to a 12-month follow-up [64]. Higher morbidity (primarily COPD exacerbations and infections) was noted in the treatment group [64]. Exhale stents are currently not a recommended treatment for lung volume reduction, and further research is needed before clinical applicability can be considered [65,66].

## 4. Investigational Therapies Focused on Mucus Hypersecretion and Inflammation

While the majority of bronchoscopic treatments for COPD are aimed primarily at reducing lung volume and therefore hyperinflation, a subset of patients have a predominantly chronic bronchitis phenotype characterized by airway inflammation, mucus hypersecretion, and resultant productive cough and dyspnea. Chronic bronchitis is classically described as chronic cough and sputum production for ≥3 months in two successive years, but there is significant variation in definition across study populations [65]. The mucus overproduction of chronic bronchitis predominantly emanates from goblet cells in first- to fifth-generation airways (up to subsegmental bronchi) and are accessible to bronchoscopically directed treatments [66].

### 4.1. Targeted Lung Denervation

Targeted lung denervation (TLD) is a novel bronchoscopic treatment aimed at attenuating parasympathetic overactivity by disrupting peribronchial vagal innervation of the lung in order to reduce bronchoconstriction and mucus hypersecretion. The procedure is performed under general anesthesia with bronchoscopic and fluoroscopic guidance. Radiofrequency energy is delivered via a double-cooled catheter (Nuvaira, Minneapolis, MN, USA) in order to produce a narrow band of ablation around the main bronchi while minimizing the effect to the inner surface of the airway. Targeted nerve fibers are disconnected from their proximal segments due to thermal injury, and subsequent wallerian degeneration degrades distal fibers out to peripheral endings along small airways with persistent cessation of acetylcholine release [67]. Early studies in TLD demonstrated both safety and feasibility, defined optimal dosing, and described adverse events [67,68,69]. Respiratory (pneumonia or COPD exacerbation) and gastrointestinal (impaired gastric emptying or gastritis) were the most commonly reported adverse events [67,69]. The AIRFLOW-2 trial (n = 82) was a multicenter, 1:1 randomized, sham bronchoscopy-controlled trial conducted in patients with symptomatic COPD (mMRC ≥ 2 or CAT > 10) with FEV1 30–60% predicted with a primary endpoint of safety (rate of respiratory adverse events at three and six months after randomization) [70]. The TLD group (n = 41) experienced fewer respiratory adverse events (defined as AECOPD, worsening bronchitis, dyspnea, wheezing, tachypnea pneumonia, other respiratory infection, or respiratory failure requiring therapeutic intervention) at the prespecified time points, but overall rates of respiratory events were similar between the two groups with a trend towards increased gastrointestinal effects in the TLD group. Two-year outcomes from AIRFLOW-2 demonstrated lengthened time to first COPD exacerbation in the TLD arm but no significant difference in lung function or SGRQ scores [71]. An ongoing multicenter sham bronchoscopy-controlled trial (AIRFLOW-3) aims to evaluate the efficacy of TLD to reduce moderate or severe COPD exacerbations with optimal medical therapy compared to medical therapy alone [72]. Additional trials are needed before TLD can be considered as a therapeutic option outside of clinical trials.

### 4.2. Bronchial Rheoplasty

RheOx^®^ bronchial rheoplasty (Gala Therapeutics, San Carlos, CA, USA) delivers short bursts of high-frequency electrical energy to the airway epithelium and submucosal tissue layers in order to target goblet cells. The procedure is generally performed in two separate treatments (one lung per treatment) with one month in between. Treatment is delivered from second- to seventh-generation airways. A multicenter single-arm clinical trial (n = 30) demonstrated significant improvements in CAT and SGRQ scores with no change in lung function parameters at 3 and 12 months [73]. A prospective multicenter sham bronchoscopy-controlled clinical trial examining safety and effectiveness of bronchial rheoplasty is ongoing [74].

#### Metered Cryospray and Balloon Deobstruction

Metered Cryospray (RejuvenAir, CSA Medical, Lexington, MA, USA) and balloon deobstruction (Rezektor Balon, Istanbul, Turkey) are two additional bronchoscopic treatment modalities specific to chronic bronchitis. Both are intended to destroy hyperplastic goblet cells via freezing and mechanical disruption, respectively [75,76]. All three of these modalities are in the very early phase of research and development, having demonstrated quality-of-life improvements but little effect on cough or sputum production [66]. The lack of consensus definition of chronic bronchitis has complicated selection of a surrogate end point for treatment efficacy. Further research is needed to examine specific changes in chronic bronchitis symptoms and evaluate durability of treatment effects. 

## 5. Conclusions

Bronchoscopic lung volume reduction is a minimally invasive procedure that offers clinical benefit comparable to lung volume reduction surgery while avoiding the substantial morbidity and longer hospital stays associated with the invasive surgical approach [13,77]. Ongoing studies in BLVR should prioritize refined patient selection and reduction of complications in order to optimize patient outcomes. Hyperinflation (defined as TLC ≥ 100%) was used as an inclusion criterion in several trials (NETT, EMPROVE, and LIBERATE), but no existing trials have evaluated whether patients with dynamic hyperinflation, a frequent source of activity limitation in COPD, could benefit from BLVR. Static hyperinflation also contributes to dyspnea in patients with COPD, and additional research is to explore the physiology of hyperinflation in patients that qualify for BLVR. Additionally, further study is needed to define the optimal bronchoscopic lung volume technique for patients who lack fissure integrity or exhibit collateral ventilation, as well as to establish longer-term clinical outcomes data.

## Figures and Tables

**Table 1:** Summary of clinical endpoints of randomized control trials of BLVR with EBV. Results shown as between-group differences when reported.

Trial	Trial Characteristics	Fissure Integrity and Heterogeneity	Follow-Up	FEV1 (mL) Change	FEV1 (%) Change	6MWT Change	SGRQ Change
VENT (2010)	Multicenter prospective RCT (n = 321)	Not specified	6 months	NR	+16.2%	+7.7%	NR
STELVIO (2015) ^	Prospective RCT (n = 68)	Enrolled CV patients	6 months 12 months (n = 64)	+140 NR	+17.8% +17%	+74 m +61 m	−14.7 −11
BeLieVer-HiFi (2015)	Single-center, double-blind, sham-controlled RCT (n = 50)	Targeted heterogeneous patients	3 months	+30	+5.9%	+22 m	−0.8 *
IMPACT ^ (2016)	Prospective multicenter RCT (n = 93)	Targeted homogenous patients	3 months	+120	+16.9%	+40 m	−7.6
TRANSFORM ^ (2017)	Prospective multicenter RCT (n = 97)	Targeted heterogenous, CV patients	3 months	+230	+29.3%	+78.7 m	−6.5
LIBERATE (2018)	International multicenter RCT (n = 190)	Targeted heterogenous, CV patients	12 months	+106	+18%	+39.3 m	−7.05
REACH (2019)	Prospective multicenter unblinded RCT (n = 107)	Targeted heterogenous, CV patients	3 months	+101	NR	+19.7 m *	−7.19 *
EMPROVE (2019)	International prospective RCT (n = 172)	Targeted heterogenous, CV patients	6 months	+101	NR	+6.9 m *	−13

^: Intention to treat analysis results reported; *: non-significant compared to control group; CV: collateral ventilation; RCT: randomized controlled trial; NR: not reported.

**Table 2:** Complications of BLVR among the seminal trials. Results displayed as absolute event rates.

Trial	Duration of Follow-Up	Pneumothorax	COPD Exacerbation	Pneumonia	Respiratory Failure	Device-Related Deaths
VENT (2010)	90 days	4.2%	9.3%	3.3%	1.4%	<1%
STELVIO (2015)	6 months	18%	12%	6%	NR	1.5%
BeLieVer-HiFi (2015)	90 days	8%	64%	8%	NR	8%
IMPACT (2016)	3 months	25.6%	16.3%	0	2.3%	0
TRANSFORM (2017)	30 days	20%	4.6%	4.6%	NR	0
LIBERATE (2018)	45 days	26.6%	7.8%	<1%	1.6%	3.1%
REACH (2019)	3 months	7.6%	19.7%	1.5%	NR	0
EMPROVE (2019)	6 months	32 events *	16.2%	8.9%	2/7%	0

*: Absolute number of pneumothoraces as multiple occurred in single patient in some cases; NR: not reported.

## Data Availability

No new data were created or analyzed in this study. Data sharing is not applicable to this article.
